# Acclimatization of *Rhizophagus irregularis* Enhances Zn Tolerance of the Fungus and the Mycorrhizal Plant Partner

**DOI:** 10.3389/fmicb.2018.03156

**Published:** 2018-12-18

**Authors:** Van Cuong Bui, Philipp Franken

**Affiliations:** ^1^Leibniz-Institute of Vegetable and Ornamental Crops, Großbeeren, Germany; ^2^Institute of Environmental Technology, Vietnam Academy of Science and Technology, Hanoi, Vietnam; ^3^Plant Physiology Department, Humboldt University of Berlin, Berlin, Germany

**Keywords:** acclimatization, arbuscular mycorrhiza, gene expression, heavy metal tolerance, root organ culture

## Abstract

Arbuscular mycorrhizal (AM) fungi confer heavy metal tolerance to plants, but this characteristic differs between different AM fungal strains. We tested the hypotheses if acclimatization of an AM fungus to Zn stress is possible and if this leads also to higher Zn tolerance of mycorrhizal plants. The AM fungus *Rhizophagus irregularis* was acclimatized in root organ cultures (*Daucus carota* L.) to Zn resulting in an acclimatized (Acc+) strain. The non-acclimatized (Acc-) strain remained untreated. Fungal development and RNA accumulation of a set of stress-related genes were analyzed in root organ cultures and the capacity of conferring Zn tolerance to maize plants was investigated in pot cultures. Development of Acc+ strain was significantly higher than Acc- strain, when strains were grown in Zn-enriched root organ cultures, whereas the growth of the Acc+ strain was reduced on normal medium probably due to a higher Zn demand compared to the Acc- strain. RNA accumulation analyses revealed different expression patterns of genes encoding glutathione S-transferase (*RiGST*), superoxide dismutase (*RiSOD*) and glutaredoxin (*RiGRX*) between the two strains. Plants inoculated with the Acc+ strain showed higher biomass and lower Zn content than those inoculated with the Acc- strain. The results showed that *R. irregularis* can be acclimatized to increased amounts of Zn. This acclimatization leads not only to improved fungal development in Zn-stress conditions, but also to an increase of mycorrhiza-induced Zn tolerance of colonized plants.

## Introduction

Arbuscular mycorrhizal (AM) fungi have received much attention due to their widespread occurrence in the soils of terrestrial ecosystems (Schüßler and Kluge, 2001). They form a mutualistic symbiosis with more than 80% of vascular plants (Smith and Read, 2008). It is assumed that AM fungi are able to acquire mineral nutrients more efficiently than root hairs and to supply it to colonized plants leading to enhanced nutrient uptake of their hosts (Marschner and Dell, 1994). In turn, they receive photosynthates that are produced by plants (Rich et al., 2017; Keymer and Gutjahr, 2018).

Besides having beneficial effects on plant nutrition, AM fungi also confer tolerance to abiotic stresses (Al-Karaki, 2006; Porcel et al., 2006; Aroca et al., 2007; Hildebrandt et al., 2007; Evelin et al., 2009; Miransari, 2010). Additionally, numerous studies have demonstrated that mycorrhizal plants can increase their capacities to tolerate heavy metal (HM) contaminations in soils (Hildebrandt et al., 1999; Gaur and Adholeya, 2004; Wang et al., 2005; Chen et al., 2007). In this regard, field and pot experiments showed that AMF strains originating from HM-polluted soils conferred higher HM tolerance to colonized plants than AMF strains from non-polluted soils (Díaz et al., 1996; Hildebrandt et al., 1999). Several research groups showed that this was accompanied by higher HM tolerance by the fungi themselves (Weissenhorn et al., 1994; del Val et al., 1999). Proposed mechanisms used by AM fungi to deal with HM stress involved avoidance strategy to prevent the presence of HM in the cytoplasm. AM fungi can reduce the bioavailability of HM by excreting compounds, such as glomalin, which can sequester large amounts of HMs in soil (Purin and Rillig, 2007; Cornejo et al., 2008). Moreover, AM fungi are able to bind HM to their cell walls limiting HM transport to plant tissues (Joner et al., 2000; Christie et al., 2004). HMs still passing through such these external barriers can be detoxified inside fungal cells by chelating agents, like glutathione or metallothionein, and subsequent disposal of chelated complexes are accomplished by the vacuole or by pumping them out of the cells (Binz and Kägi, 1999; Sheehan et al., 2001). Despite it has been observed that AM fungi isolated from elevated HM conditions are more tolerant to HM stress than those who are not (Weissenhorn et al., 1994; del Val et al., 1999), the molecular and/or genetic mechanism that drives this phenomenon is poorly understood. Adaptation processes could play a role, but providing genetic evidences is difficult, because such long-term evolutionary processes are accompanied by many sequence alterations making it nearly impossible to find those which are responsible for HM tolerance. Particular investigations showed that the achieved HM tolerance of AM fungi was not stable. The level of HM tolerance decreased or even got lost if AM fungi were kept in HM-free substrate, and this decrease subsequently affected the HM tolerance of host plant (Weissenhorn et al., 1994; Malcová, 2003). This opened the possibility to compare genetically related strains and, moreover, raised the question if HM tolerance can only be lost or also gained.

Identification and expression analyses of AM fungal genes putatively encoding functions involved in HM tolerance were mostly conducted in root organ cultures. This showed that such genes can be induced by various HMs such as Pb, Cu, Zn, or Cd and also by oxidative agents like paraquat (González-Guerrero et al., 2005, 2007, 2010a,b; Waschke et al., 2006; Benabdellah et al., 2009a,b). It has not been studied if expression patterns differ between AM fungal strains which exhibit different degrees of HM tolerance. Such a comparative study could result in the identification of particular mechanisms, because it could distinguish genes, which are directly regulated as a consequence of HM stress, from genes which might express products involved in the processes leading to tolerance. It is, however, important that AM fungal strains chosen for such a study are highly related concerning their genetic background.

In this study, we analyzed two hypotheses. The first hypothesis says that AM fungi are able to gain heavy metal tolerance if they are cultivated in high HM condition. To test this hypothesis, we used root organ cultures to acclimatize the AM fungus *Rhizophagus irregularis* to high Zn concentrations. Acclimatization was monitored and concluded successful or not by observing AM fungal development *in vitro.* The putative molecular background of the acclimatization process was analyzed by expression analyses of a set of AM fungal genes. The genes were selected on previous analyses of HM tolerance in AM fungi representing the three classes involved in metal homeostasis (Bolchi et al., 2011). The second hypothesis predicates that an acclimatized AM fungus with higher heavy metal tolerance can better confer tolerance to its colonized host plants. For testing this proposition, maize plants were inoculated with an acclimatized strain or its non-acclimatized counterpart, respectively. Plants were cultivated in soils harbouring different Zn amounts, and biomasses as well as Zn and P content and uptake were monitored.

## Materials and Methods

### Root Organ Cultures and Acclimatization of the Fungus

The AM fungus *R. irregularis* (DAOM 181602) was cultivated in carrot (*Daucus carota* L.) root organ cultures on Petri dishes (18 cm) containing 100 mL M-medium agar according to Bécard and Fortin (1988). Cultures were incubated at 24°C in the dark. To define the appropriate Zn concentration for acclimatization, a pre-test was conducted growing the AM fungus and its host root in different Zn concentrations (20, 40, 60, 80, 100, and 120 μM). Zn concentration at 80 μM showed a slight inhibition of fungal growth. Therefore, Zn concentration at 80 μM was chosen. For acclimatization, each 30 spores of the original strain were cultivated in two parallel linages with or without the presence of Zn (80 μM). After 10 weeks, spores of each linage were randomly selected and transferred to a new carrot root culture with or without Zn for the next generation. This process was repeated five times. The sub-strains obtained from the last cultures were called “acclimatized” (Acc+) or “non-acclimatized” (Acc-).

To examine the efficiency of the acclimatization process, new root organ cultures were inoculated with spores of the Acc+ and the Acc- strain. The test was conducted in a two compartment system (St-Arnaud et al., 1996) where one compartment contained the mycorrhizal roots (this part contains 0 or 80 μM Zn for Acc- or Acc+ respectively) from where extraradical hyphae grew into the second compartment (containing 200 μM Zn in the first test or 0 and 200 μM in the second test, respectively). After 7 weeks of cultivation, the number of spores and density of hyphae were quantified in the hyphal compartment (five counting points per plate) by the grid-line method under a binocular with 20x magnification (modified from Giovannetti and Mosse, 1980). Each setup consisted of five biological replicates.

### RNA Accumulation Analyses

The split Petri dish system was also used for RNA accumulation analyses in extraradical hyphae. Fresh root organ cultures in two compartment Petri dishes were inoculated with spores of both strains. Five weeks after start of the cultures, agar in the hyphal compartments was replaced by liquid M-medium without sucrose, and new hyphae grew into the liquid medium for 2 weeks (Waschke et al., 2006). Thereafter, hyphae were challenged by adding a Zn solution to reach a final concentration of 200 μM Zn. Extraradical hyphae were harvested after 0 h (as a control), 2, 4, 6, and 24 h exposure to Zn. Hyphae were cut with a scalpel, immediately frozen in liquid nitrogen and stored at -80°C for further analysis. RNA was extracted from hyphae using the RNeasy Plant Mini Kit including the on-column-DNase treatment (Qiagen, Hilden, Germany) according to manufacturer’s manual. The quality and quantity of the RNA was measured in the Bioanalyzer 2100 (Agilent Technologies, Boeblingen, Germany). cDNA was synthesized from 1 ng RNA as template in 20 μL final volume using the Maxima first strand cDNA synthesis kit (Thermo Fisher Scientific, Rockford, IL, United States). Synthesized cDNA was used as template for quantitative real time PCR (7500/7500 Fast real time PCR system, Applied Biosystems, Foster City, CA, United States). Each reaction contained 5 μL powers SYBR green 2× (Thermo Fisher Scientific, Rockford, IL, United States), 200 nmol/L of each forward and reverse primers (Supplementary Table S1) and 1 μL cDNA template in a final volume of 10 μL. Primer pairs were designed with the software Primer Select (GATC Biotech, Konstanz, Germany) based on genomic and transcript sequence information of *R. irregularis* (Tisserant et al., 2012, 2013). Primers spanned intron-exon boundaries to avoid the amplification of genomic DNA contamination. Primer quality was checked by PCR on total RNA in comparison with cDNA. Amplification product length was below 200 bp and a consistent annealing temperature of approximately 60°C was considered for all primer pairs to allow simultaneous analysis of reference and target gene on one plate. The qRT-PCR efficiency calculation was conducted with LinReg (Amsterdam, Netherlands). Efficiency values between 1.8 and 2.0 were accepted. Each treatment harbored three biological replicates and three technical replicates. Relative levels of transcription were calculated by using the 2^ΔCt^ (ΔCt = Ct reference gene – Ct target gene). Target and reference genes are listed in Supplementary Table S1.

### Pot Cultures

Maize (*Zea mays* L. cv. Luigi) seeds were surface sterilized with 13 % H_2_O_2_ for 3 min, washed three times with sterilized water and germinated on water agar for 1 week before transfer to pots. A phosphate-deficient loess from the C-horizon (Müller et al., 2013) was sterilized in an oven at 85°C for 24 h, cooled down to room temperature for another 24 h, and again heated to 85°C for 24 h. Sterilized soil was mixed with nutrients for mycorrhization experiments according to Müller et al. (2013). Half of the pots received ZnSO_4_ to obtain a final concentration of 800 mg Zn/kg dry soil. Soil was incubated for 10 days after addition of nutrients before use. Each pot (height 15 cm, Ø 19 cm), with five biological replicates per treatment, contained 2.5 kg soil.

AM fungal spores were separated from solid M-medium according to Doner and Bécard (1991). The spores were washed three times with distilled sterilized water, and around 100 ± 5 spores were applied to roots of 1 week old maize seedlings of similar size. The water used for conducting the last washing step of spores was passed through filter paper (Whatman, Schleicher and Schuell, Germany) and used for the non-mycorrhizal controls.

### Mycorrhization Assessment and Mineral Nutrient Analysis

Plants were harvested after 2 months of cultivation, and shoots and roots were divided. Roots were separated from soils by washing, and a subsample around 1 g of fresh roots was collected for mycorrhization assessment following the method of Trouvelot et al. (1986) with the modification that vesicle abundance was also calculated according to the scheme for arbuscule abundance. Shoots and roots were dried at 65°C for 3 days. After measuring the dry weights, shoots and roots were ground separately using a centrifugal rotor mill at 1,800 rpm (ZN 200, Retsch, Germany). Around 200 mg of ground shoot and root samples were digested with 5 mL 65% HNO_3_ and 3 mL H_2_O_2_ 30% in a microwave apparatus (Mars, CEM Corporation, Germany). The digested solution was made up to volume (50 mL) with double distilled water. P and Zn were analyzed using ICP-OES (iCAP DUO 6000, Thermo Scientific GmbH, Germany) after filtration through filter paper (Whatman, Schleicher and Schuell, Germany).

The hyphal compartments inserted to each pot were prepared following Neumann and George (2005). To extract the extraradical hyphae, the content of the fungal compartments was mixed with deionized water in a bowl. After the glass beads and fungal hyphae had settled to the bottom of the container, the suspended particles were poured through a 40 μm sieve. Tap water was used to wash remaining substrate particles through the sieve, leaving only the hyphae. The hyphae were then freeze-dried at -30°C for 4 days. After measuring the dry weights, hyphae were used for mineral analysis (P and Zn) as described above for plant tissues analysis.

### Statistical Analysis

The statistical analysis was carried out with the software Statistica (version 12, Tulsa, OK, United States). The variables were tested for normality of distribution (Kolmogorov–Smirnov test). The variables which were not normal distributed or given in percentage values were transformed by logarithm or arcsine, respectively. Two-way analysis of variance (ANOVA) was used for data analyses. Tukey HSD was performed at *p* = 0.05 in case of significant interaction between factors. If no significant interaction between factors was detected, the mean values were pairwise compared (between control and Zn treated samples) by Student’s *t*-test at *p =* 0.05. All data are shown as mean values with standard deviations (SD).

## Results

### Acclimatization of *Rhizophagus irregularis* in Root Organ Cultures

**FIGURE 1:**
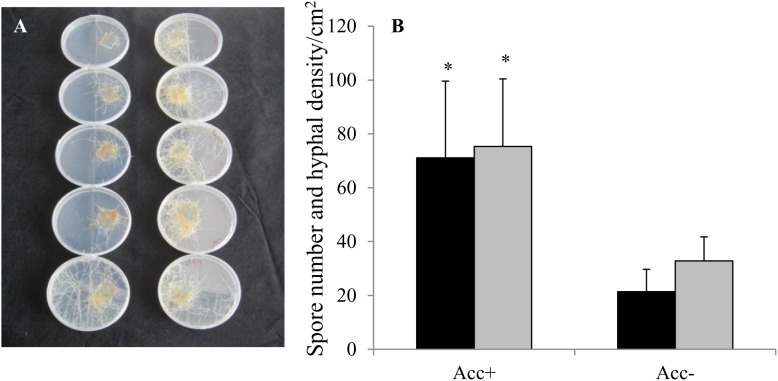
*Rhizophagus irregularis* strains acclimatized (Acc+) and non-acclimatized (Acc-) in carrot root organ cultures under Zn stress. Thirty spores of each fungal strain were placed onto 2 cm^2^ of carrot roots in compartmented Petri dishes containing M-medium with 200 μM Zn. After 7 weeks of growth **(A)**, spore numbers (dark columns) and hyphal densities (light columns) were quantified in the second compartment by grid-line method **(B)**. Mean values and standard deviations are shown. Asterisks indicate significant differences between Acc+ and Acc- strain (Student’s *t*-test, *p* = 0.05, *n* = 5).

In the first test to confirm the efficiency of acclimatization process, the results showed that the Acc+ strain developed 2.3 times more hyphae and 3 times more spores at 200 μM Zn than the Acc- strain (Figures 1A,B).

**FIGURE 2:**
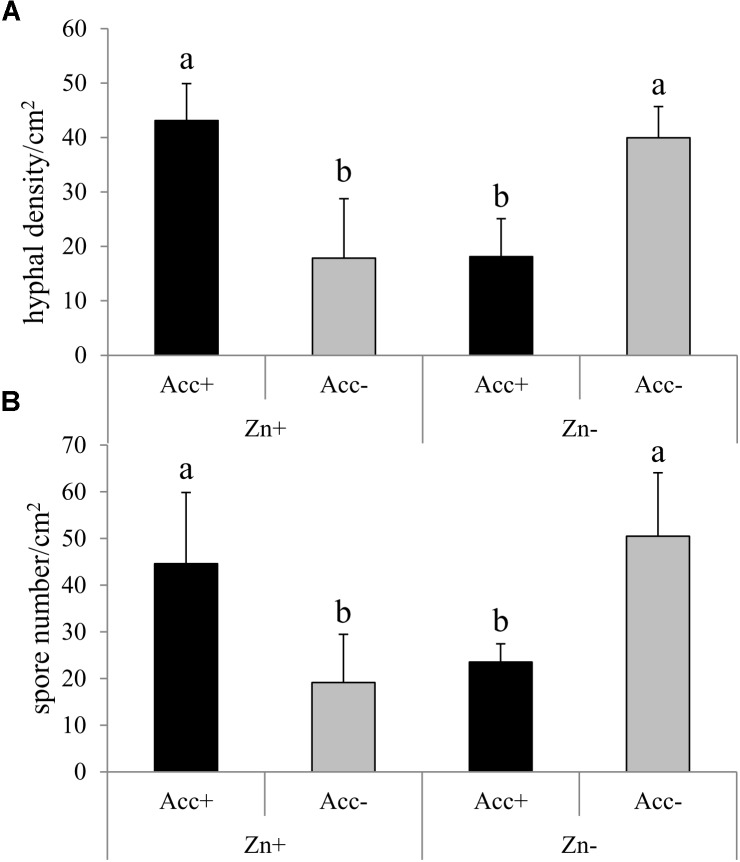
Hyphal density **(A)** and spore number **(B)** of *R. irregularis* Acc+ and Acc- strains in M-medium with (Zn+) or without 200 μM Zn (Zn-). Experiment was conducted and data obtained as described in the legend of Figure 1. Two way ANOVA (*p* = 0.05, *n* = 5) showed a significant interaction between the factors “acclimatization” and “Zn.” Different letters indicate significant differences according to *post hoc* Tukey HSD test.

After the first test had confirmed the efficiency of the acclimatization process for enhancing fungal tolerance to high Zn concentrations (Figure 1), a second independent test was conducted to investigate the performance of the two strains at a Zn concentration of 200 μM compared to no Zn treatment. In this second test, hyphal density and spore number of the Acc+ and the Acc- strain significantly differed depending on the addition of Zn to the medium (Figure 2). Results were consistent with the previous test as hyphal density and spore number of Acc+ strain was significantly higher than of the Acc- strain when both were grown in Zn-enriched medium. An opposite pattern was, however, observed in the basic medium where development of the Acc+ strain was reduced compared to the Acc- strain (Figure 2).

### Fungal Gene Expression in Root Organ Cultures

**FIGURE 3:**
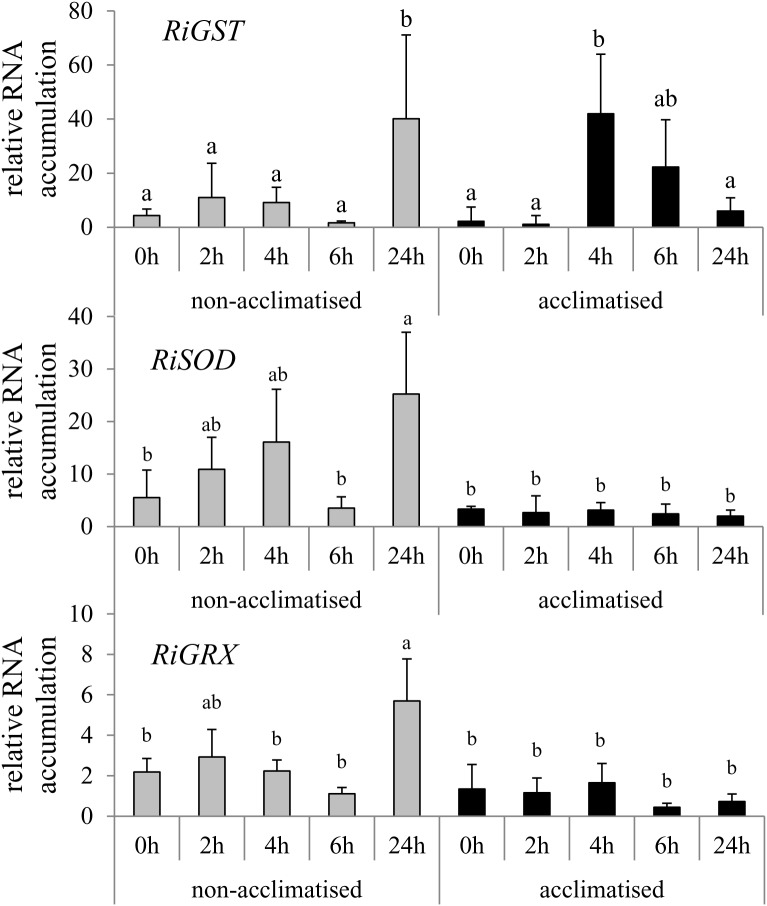
Time course analysis of gene expression in hyphae of acclimatized and non-acclimatized *R. irregularis* strains in root organ cultures under Zn stress. Hyphae were harvested after 0, 2, 4, 6, and 24 h exposure to 200 μM Zn stress. RNA accumulation of genes was quantified by qRT-PCR using specific primers and the *RiTef* gene for calibration. The relative levels of transcription were calculated by using the 2^ΔCt^ method. Mean values and standard deviations are shown. Two way ANOVA (*p* = 0.05, *n* = 3) showed significant interaction of the two factors “time of exposure” and “acclimatization” for the gene expression of *RiGST*. Different letters indicate significant differences according to *post hoc* Tukey HSD test.

To get insight into the molecular basis of the acclimatized *R. irregularis* in high Zn concentrations, RNA accumulation of a selection of genes was investigated which have been described to be induced under HM stress (Supplementary Table S1). The results for only those genes are shown which possessed significant influence of at least one of the two factors “acclimatization” or “time of exposure” or significant interaction between the two factors (Figure 3).

The quantitative RT-PCR analysis revealed that *RiZnT*, *RiABC, RiPDX,* and *RiMT* encoding a Zn transporter, an ATP binding cassette transporter, a pyridoxal 5’-phosphate synthase or a methallothionein showed changes in RNA accumulation neither in the Acc+ nor in the Acc- strain throughout the time course of the experiment (data not shown). The genes *RiGRX* (glutaredoxin) and *RiSOD* (superoxide dismutase) of the Acc- strain were, however, induced already 2 or 4 h after applying Zn stress. Temporary down-regulation of the expression of the two genes in Acc- strain was observed after 6 h and a strong up-regulation again at the 24 h time point. Induction of *RiGRX* and *RiSOD* was not observed in the Acc+ strain (Figure 3). In contrast, *RiGST* (glutathione S-transferase), showing a similar RNA accumulation pattern in the Acc- strain as *RiGRX* and *RiSOD*, was highly induced already after 4 h of Zn exposure in the Acc+ strain. Thereafter RNA accumulation declined and reached a value at 24 h, which was similar to the amount of expressed transcripts at the 0 h of the experiment.

### Mycorrhization, Plant Growth, and Nutrient Uptake

In order to test if increased Zn tolerance can be transferred from the acclimatized AM fungus to the mycorrhizal plant (second hypothesis), a pot culture experiment was conducted with maize as host plant. Plants were inoculated with spores of *R. irregularis* Acc+ and Acc- strains and cultivated in a phosphate-deficient soil fertilized with a standard fertilizer or with fertilizer harboring high Zn concentrations. Appropriate Zn concentrations under the present conditions were defined before in a preliminary experiment (data not shown). Two-month old plants were harvested, and the mycorrhization of the roots, shoot and root biomasses, as well as P and Zn content of the shoots and roots were analyzed. Uptake of the elements was calculated by multiplying contents with biomasses.

**FIGURE 4:**
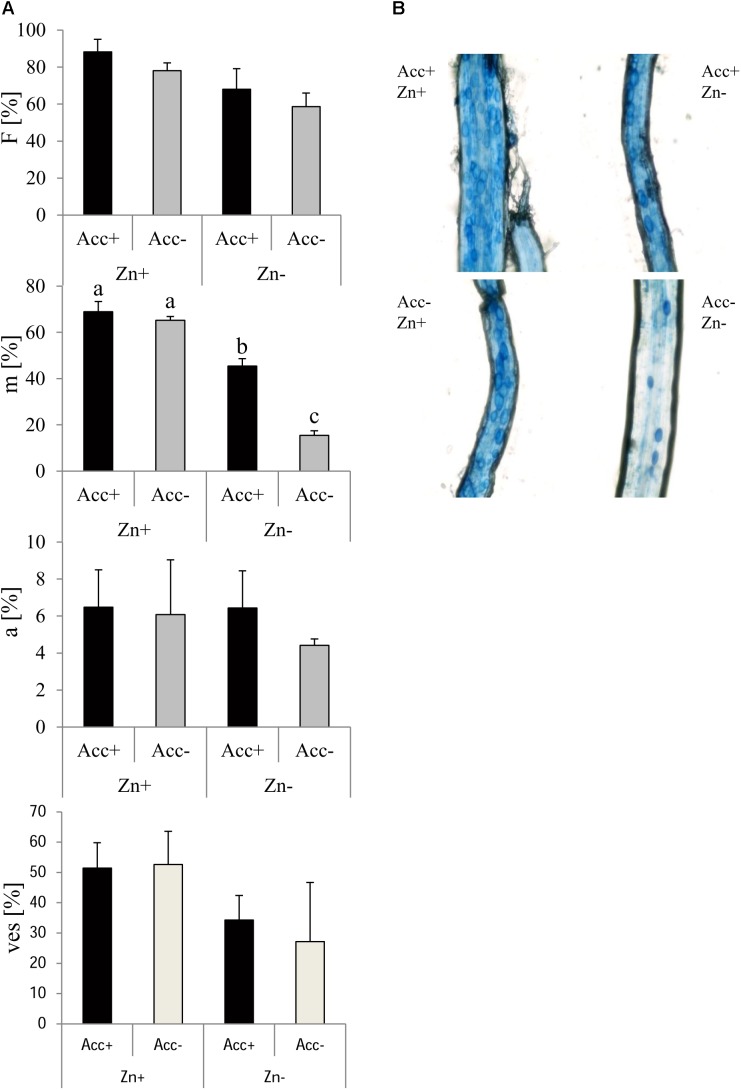
Mycorrhization of maize plants by *R. irregularis* Acc+ and Acc- strain in Zn-enriched (Zn+) and normal (Zn-) soils. Mean values and standard deviations of mycorrhization parameters are shown **(A)**. Two way ANOVA (*p* = 0.05, *n* = 5) showed a significant effect of factors “Zn” and “acclimatization” on infection frequency (F) but no interaction. Relative mycorrhization (m) was influenced by both factors and their interaction. Different letters indicate significant differences according to *post hoc* Tukey HSD test. No factor affected relative arbuscular abundance (a). Relative vesicle abundance (ves) was influenced by “Zn” as can be seen in the microscopic pictures **(B)**, but not by “acclimatization” and no interaction was detected.

Trypan blue staining and microscopy were carried out to assess mycorrhizal parameters (Figure 4). This showed at first that more root fragments harbored AM fungal structures (infection frequency, F %), when plants were grown at high Zn and when they were inoculated with the acclimatized strain. Fungal spread inside the roots (relative mycorrhization, m %) was generally higher in soils containing high amounts of Zn. In addition, the acclimatized strain showed increased colonization of the roots, but this was only significant at normal soil (no Zn addition). Relative arbuscule abundance (a %) was not affected, but relative vesicle abundance was significantly higher in roots of plants grown at high Zn. Differences of vesicle abundance (ves %) between Acc+ and Acc- strain were not detected.

**FIGURE 5:**
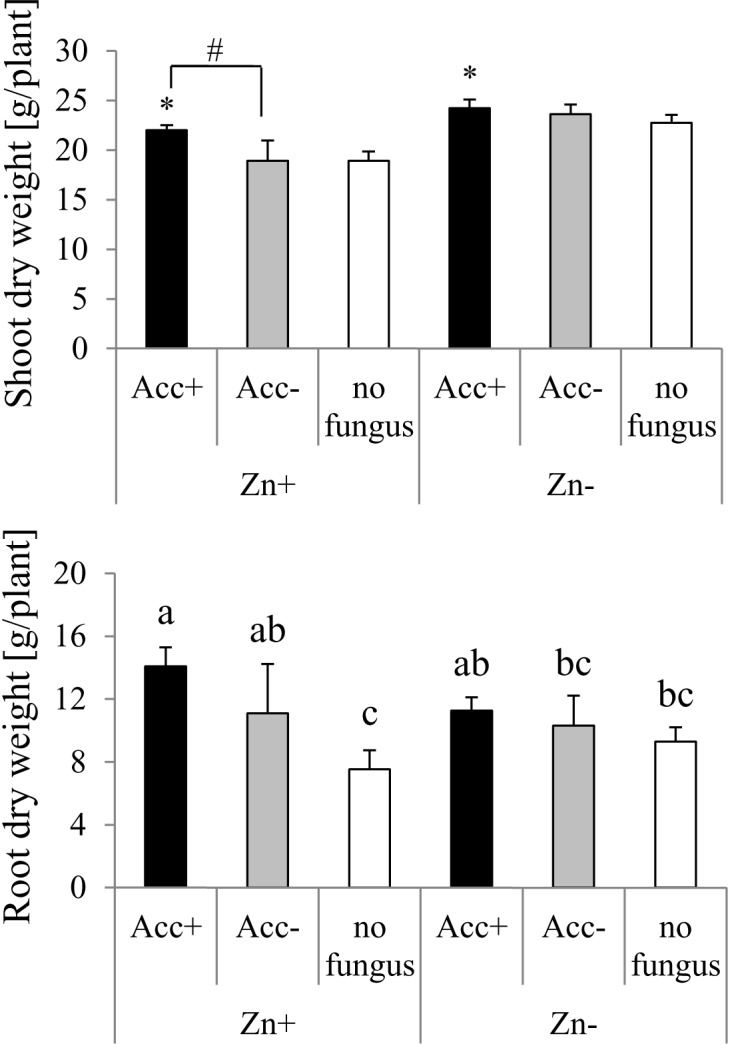
Dry weights of maize shoots and roots. Treatments are indicated as in Figure 4. Mean values and standard deviations are shown. Two way ANOVA (*p* = 0.05*, n* = 5) revealed interaction between factors “Zn” and “acclimatization” for root dry weight and significant differences between treatments are indicated by different letters according to *post hoc* Tukey HSD test. Shoot dry weight is significantly influenced by both factors, but interaction of factors could not be detected. Asterisks indicate significant differences of shoot dry weight between inoculated plants and non-inoculated controls, whereas hashtag indicates significant difference between plants inoculated with Acc+ and Acc- strain (Student’s *t*-test, *p* = 0.05*, n* = 5).

The negative effect of high Zn amounts in soil was reflected by reduction of shoot biomass of plants (Figure 5). Inoculation with the Acc+ strain enhanced biomass production compared to controls. In soils containing high Zn amounts, a significant difference was also detected between plants inoculated with Acc+ and Acc- strains. In contrast to shoot biomass, root biomass was generally not affected by Zn addition (Figure 5). However, mycorrhization significantly increased root biomass as compared to non-mycorrhization at high Zn amounts, but a significant difference between both strains was not observed. In soils without added Zn, root production was similar among non-inoculated plants and plants colonized by the two fungal strains (Figure 5).

**FIGURE 6:**
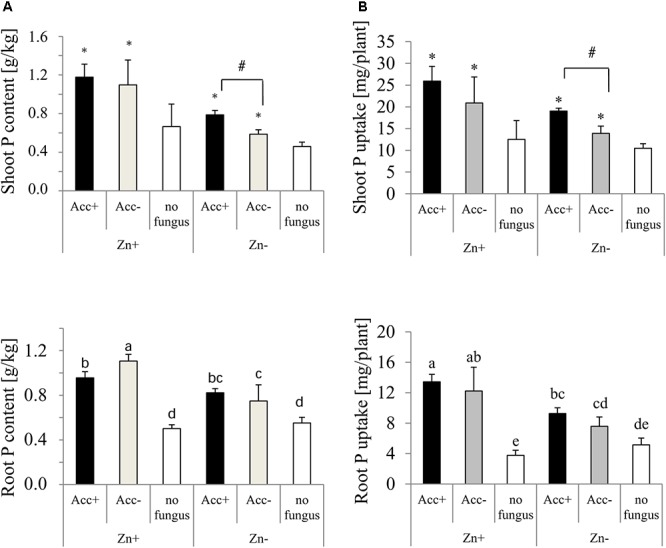
P content **(A)** and uptake **(B)** of shoots and roots of maize plants. Treatments are indicated as in Figure 4. Mean values and standard deviations are shown. **(A)** Two-way ANOVA (*p* = 0.05; *n* = 5) analysis of root P content showed significant interactions between the factors “Zn” and “acclimatization.” In shoots, P content is significantly influenced by both factors, but no interaction was observed. **(B)** Two-way ANOVA (*p* = 0.05; *n* = 5) analysis of P uptake of roots showed interactions between the factors “Zn” and “acclimatization.” P uptake of shoots is significantly influenced by both factors, but no interaction was observed. In case of interaction, the significant differences between treatments are indicated by different letters according to *post hoc* Tukey HSD test. In case of no interaction, asterisks indicate significant differences of P content and uptake between inoculated plants and non-inoculated controls, whereas hashtag indicates significant difference of plants inoculated with Acc+ and Acc- strain (Student’s *t*-test, *p* = 0.05, *n* = 5).

P was generally more present in shoots than in roots (Figure 6). Both, P content and uptake in shoots and roots were significantly enhanced by the presence of the two AM fungal strains, and this enhancement was further increased in plants where the fertilizer contained high Zn concentrations (Figure 6). Significant differences between plants inoculated with Acc+ and plants with Acc- were observed when the plants were grown in soils without additional Zn. In roots, however, P content was decreased in plants colonized by the Acc+ strain compared to Acc- colonized plants, if they were grown in Zn-enriched soils. P uptake in roots was not different between inoculated plants in both soil conditions.

**FIGURE 7:**
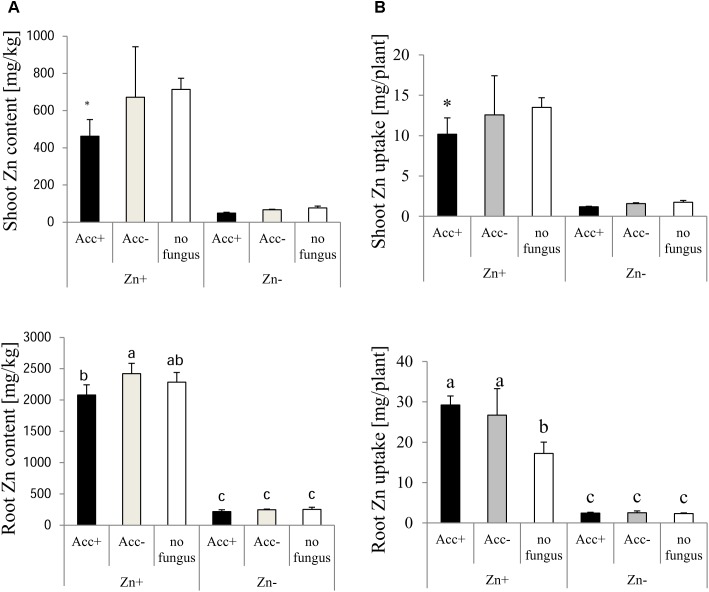
Zn content **(A)** and uptake **(B)** of shoots and roots of maize plants. Treatments are indicated as in Figure 4. Mean values and standard deviations are shown. **(A)** Two-way ANOVA (*p* = 0.05; *n* = 5) analysis of Zn content in roots showed interactions between the factors “Zn” and “acclimatization.” Zn content of shoots was significantly influenced by Zn, but not by acclimatization, and no factor interaction could be observed. **(B)** Two-way ANOVA (*p* = 0.05; *n* = 5) analysis of Zn uptake in roots showed significant interactions between the factors “Zn” and “acclimatization.” In case of interaction, the significant differences between treatments are indicated by different letters according to *post hoc* Tukey HSD test. In case of no interaction, asterisks indicate significant differences of Zn content and uptake between inoculated plants and non-inoculated controls, whereas hashtags indicate significant differences between plants inoculated with Acc+ or Acc- strain (Student’s *t*-test, *p* = 0.05, *n* = 5).

Opposite to P, Zn was allocated more to roots than to shoots (Figure 7). No differences were observed in plants fertilized with normal Zn amounts. When additional Zn was added to the soil, however, the impact of the AM fungi became obvious. Zn content and uptake in shoots tended to decrease in inoculated plants compared to non-inoculated plants and this became significant in plants inoculated with the Acc+ strain. Zn content in roots was not affected by mycorrhization but the uptake was higher in roots of all mycorrhizal plants compared to non-mycorrhizal controls (Figure 7).

**FIGURE 8:**
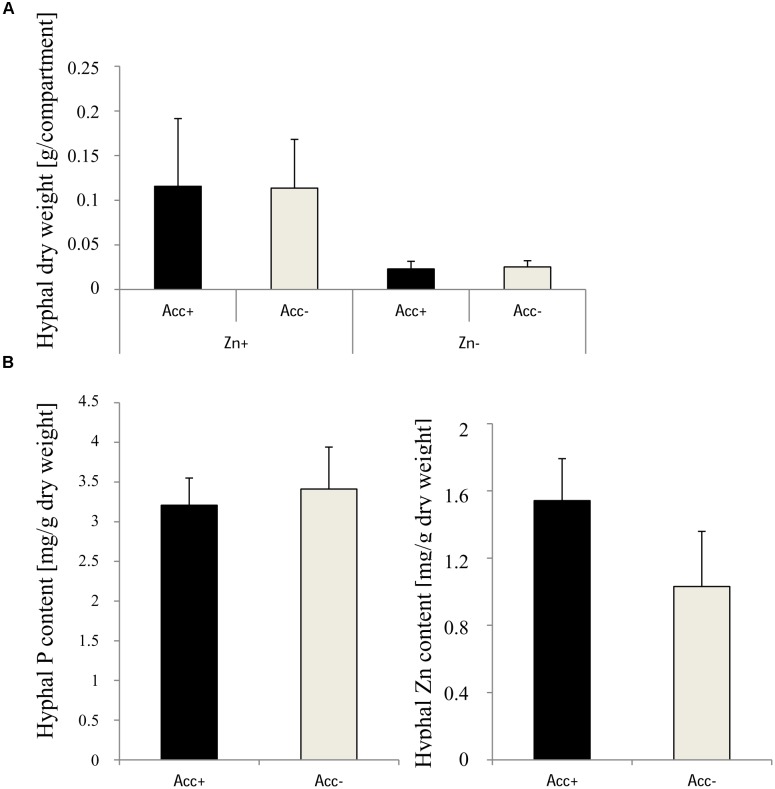
**(A)** Dry weights of extraradical hyphae of Acc+ and Acc- strains harvested from compartments. Treatments are indicated as in Figure 4. Mean values and standard deviations are shown. Two-way ANOVA (*p* = 0.05; *n* = 5) showed significant influence of the factor “Zn” on hyphal dry weight of the two fungal strains, but no influence of the factor “acclimatization” and an interaction between the factors was not detected. **(B)** Content of P and Zn in extraradical hyphae extracted from compartments in Zn-polluted (Zn+) soils. No significant differences were detected between Acc+ and Acc- strains (Student’s *t*-test, *p* = 0.05, *n* = 3).

To evaluate the capacity of extraradical hyphae to bind zinc, two hyphal compartments were installed in each pot to gather extraradical hyphae from soil. The collected hyphae were freeze dried and used for the analysis of biomass and of P and Zn content (Figure 8). Dry weights of hyphae were significantly higher in compartments of pots harboring high zinc concentration, but acclimatization had no influence. P and Zn content could only be measured in the hyphae harvested from the Zn-enriched soil. While Zn content tended to be higher in the Acc+ hyphae, no difference between the two fungal strains was detected for P (Figure 8).

## Discussion

### Acclimatization of an AM Fungus in Root Organ Cultures

This study confirms our first hypothesis: AM fungi are able to gain HM tolerance when they were grown in high HM condition. Because spores were randomly selected from one to the next cycle, we assume that the acclimatization process took place in the whole population of spores. Weissenhorn et al. (1994) proposed that higher HM tolerance observed in AM fungi is most likely related to phenotypic plasticity rather than to a change in the genotype. One possibility is priming of the AM fungal tolerance system, and this priming results in a more effective response to HM stress (Ernst, 1996). If this is the case, the growth of Acc+ strain should not be reduced when it is introduced back to normal condition. In this study, growth of the Acc+ strain was, however, suppressed at normal Zn concentration compared to the Acc- strain. The basic medium seemed to be unable to support growth of the acclimatized strain due to a Zn-deficiency. The “need hypothesis” supposed by Antosiewicz (2014) might explain this observation. After a time growing under elevated metal condition, fungi can adjust their internal homeostasis concerning essential metals like Zn to a new state, and the adjustment leads to a higher demand of the metal to which the fungi have been acclimatized to. The Zn amount in basic medium may not provide sufficient Zn for the higher needs of the Acc+ strain resulting in growth inhibition. Because the process was, however, only followed with one strain, the trait of growth reduction at high Zn concentrations might be specific for this particular strain.

### Fungal Gene Expression in Root Organ Cultures

Two strains were obtained by acclimatization, and these two strains were based on the model AM fungus *R. irregularis* for which comprehensive sequence data are available (Tisserant et al., 2012, 2013). This allowed access to the molecular basis for the observed differences in Zn tolerance by conducting comparative RNA accumulation analyses. There are three main classes of metal homeostasis related genes: genes involved in metal transport, in oxidative stress defense and in metal detoxification (e.g., Bolchi et al., 2011). Members of these classes were selected, but only *RiGST*, *RiSOD* and *RiGRX* involved in oxidative stress defense responded to the Zn treatment. It is, however, clear that the selection was not very representative, and it cannot be ruled out that other genes involved in metal transport and metal detoxification than analyzed might be differentially regulated.

The temporary down-regulation, although it is not significant, of the genes at 6 h time point in the Acc- strain corresponded with the phenomenon of biphasic ROS production which was observed in a number of other studies (for review see Mittler et al., 2011). Induction of the expression levels of *RiSOD* and *RiGRX* was, however, not observed in the Acc+ strain. Several reports have shown that suppression of enzymes involved in ROS scavenging resulted in higher tolerance to biotic or abiotic stress in plants (Chamnongpol et al., 1998; Rizhsky et al., 2002; Wong et al., 2004). The authors suggested that the down regulation of the corresponding genes facilitated the production of ROS, which function as stress signaling molecules to trigger particular processes for minimizing damage to cell components. If similar processes were active in the current study, acclimatization would lead to suppression of ROS scavenger expression, which potentiates ROS accumulation resulting in a faster activation of the tolerance mechanisms. The up-regulation of the expression level of *RiGST* in the Acc+ strain at 4 h after Zn treatment mirrors this early response. Glutathione S-transferases can act directly on stress agents (like heavy metals) by conjugation to glutathione for detoxification. The metal-conjugate complex is less toxic than free ions, and the complex can be eliminated by transporting it to the vacuole or by pumping it out of the cell (Sheehan et al., 2001). The non-acclimatized strain, however, responds to ROS increase by early up-regulation of the expression levels of *GRX* and *SOD*. The response leads to reduced ROS concentrations, and ROS does not reach the threshold level to activate the early tolerance machinery. Therefore, early expression of *GRX* and *SOD* and in turn late induction of *GST* expression makes the Acc- strain more sensitive to Zn stress.

Different hypotheses exist about the organization of AM fungal genomes (Ropars and Corradi, 2015). One of these hypotheses states that AM fungi contain many nuclei, which are genetically different. Differential gene expression could be therefore based on the selective accumulation of particular nuclei during the acclimatization process. The consequence would be the expression of different alleles from one locus in the Acc+ and the Acc- strain (Ehinger et al., 2012). Further sequencing of transcripts and genomes of individual nuclei must clarify, if differential expression of the same allele or the occurrence of different genomes accompanies variations in HM tolerance. The current Acc+ and Acc- strains could be a suitable system for distinguishing between these possibilities.

### Mycorrhization of Plants

Many studies reported a reduction of mycorrhizal colonization upon heavy metal treatment (Weissenhorn and Leyval, 1995; Leyval et al., 1997; del Val et al., 1999), whereas others showed an increase (Hildebrandt et al., 1999; Audet and Charest, 2006; Turnau, 2014). In the current study, mycorrhization of maize roots by both strains was more intense in the soil with high Zn amounts (Figure 4) also reflected by increased biomasses of extraradical hyphae (Figure 8A). While arbuscule abundance was not specifically affected, the relative number of vesicles was increased. Vesicles are formed by AM fungal species belonging to the Glomeraceae (Walker, 1995) and are considered as storage organs (Smith and Read, 2008). Besides that, vesicles were reported to capture a variety of HMs (Weiersbye et al., 1999; González-Guerrero et al., 2008; Turnau, 2014). Increased formation of vesicles in the current study could lead to limited Zn translocation from roots to shoots in Zn-enriched soils. This scenario is supported by the finding that Zn content in shoots of mycorrhizal plants is decreased compared to non-mycorrhizal plants, despite the similar Zn content in roots (Figure 7). It does, however, not explain why Zn content in shoots are only reduced if roots are colonized by the Acc+ strain (Figure 7), because no significant difference in vesicle numbers between the two strains could be observed (Figure 4). A potential explanation could be that the general capacity of Zn sequestration and also by vesicles increased upon acclimatization as Zn content in extraradical hyphae of the Acc+ strain was higher than of the Acc- strain (Figure 8B). The ability of Acc+ hyphae to sequester higher Zn amounts may be a result of changes in the cell wall composition and structure during the acclimatization process. This was previously suggested by Lanfranco et al. (2002) for an ericoid mycorrhizal fungus. The authors revealed that hyphae were thicker with an increased chitin amount when the hyphae were treated with Zn.

### Plant Growth and P and Zn Uptake

Analysis of shoot biomasses confirmed also our second hypothesis: the acclimatized strain could confer its higher HM tolerance to the plant. Plants inoculated with the Acc+ strain showed higher shoot biomasses than plants colonized by the Acc- strain and, this was only evident under Zn stress (Figure 5). While plants in Zn-enriched soils generally showed lower shoot biomasses than those in normal soils, root production was not affected. Increase of the root-shoot ratio is considered as a general strategy of plants confronted with stress (Watts-Williams et al., 2013). Such a strategy could be supported by the symbiosis because root biomass of mycorrhizal plants in polluted soils was higher than those of non-mycorrhizal plants. This might explain why the two AM fungal strains increased P uptake in the normal soil to a lesser extent than in Zn-enriched soil.

Inoculation with the Acc+ strain helped plants to take up more P and also increased P transport from roots to shoots more effectively than inoculation with the Acc- strain (higher total P uptake and higher uptake in shoots than in roots; Figure 6). This ability of the Acc+ strain could be attributed to the higher tolerance to Zn stress that enables the strain to maintain symbiotic function under stress condition compared to the Acc- strain. The advantage of the Acc+ strain could be, however, observed even in soil without Zn stress. In the initial stages of colonization, plant recognizes AM fungi as pathogens (Garcia-Garrido and Ocampo, 2002), which leads to induce particular defense activities such as ROS production (Salzer et al., 1999). Acc+ strain possessing higher tolerance to oxidative stress might be able to tolerate such defense reactions more easily than the Acc- strain. This could help the Acc+ strain to perform symbiotic functions more efficiently than the Acc- strain. The higher ability of the Acc+ strain to tolerate more oxidative stress did, however, not abolish its ability to recognize ROS as signals during the interaction. Such recognition is important as ROS signaling was shown to be crucial for arbuscule formation inside root cortex cells (Belmondo et al., 2016).

Inoculation by the two fungal strains did not influence the Zn content in roots, but uptake was increased due to the higher biomasses (Figure 7). The amounts were, however, not raised in the shoots of mycorrhizal plants or even reduced in case of plants inoculated with the Acc+ strain. Shoot to root ratio for Zn uptake of non-mycorrhizal plants reached a value of 0.78, the values in plants inoculated with the Acc- or Acc+ strain were 0.47 and 0.34, respectively. Hence, non-mycorrhizal plants transported 1.5–2.0 times more Zn from roots to shoots than mycorrhizal plants implicating that Zn can be retained by the intraradical fungal hyphae as discussed above. This binding of Zn seems to be more efficient in the acclimatized Acc+ strain also reflected by the higher Zn content in the extraradical hyphae (Figure 8B).

## Conclusion

The AM fungus *R. irregularis* can be acclimatized to increased Zn tolerance but at the expense of growth under conditions with normal Zn conditions. The differences between Acc+ and Acc- strains detected in root organ cultures were, however, not observed in pot cultures. The development of extraradical hyphae and mycorrhization parameters did not correlate with *in vitro* hyphal development and spore production. The fundamental differences between *in vitro* conditions with artificial nutrition and pot culture conditions with natural complex components in soil might alter the performance of the fungi. The expression of genes encoding ROS scavenging enzymes was suppressed in the acclimatized strain. This could lead to accelerated ROS production, and in turn to an earlier activation of the tolerance system reflected by advanced RNA accumulation of *RiGST*. Such an increased Zn tolerance of the Acc+ strain results in higher amounts of Zn sequestered by fungal hyphae and in turn to less Zn allocation to plant shoots, which allowed the plant to form higher shoot biomasses. Why the P uptake was increased in mycorrhizal plants with the Acc+ strain even in normal soil could be an interesting subject for future research. In addition, this study has interesting implications for application. Feldmann and Grotkass (2002) proposed directed AM fungal inoculum production in order to supply tailor-made inocula. The current report shows that this is principally possible, at least in case of mycorrhiza-induced Zn tolerance. Specific gene expression patterns accompanied with acclimatization processes as observed in the current study could provide tools for quality control during the production of particular AM fungal inocula.

## Author Contributions

VB and PF designed the research, interpreted the data and wrote the manuscript. VB performed the research and analyzed the data.

## Conflict of Interest Statement

The authors declare that the research was conducted in the absence of any commercial or financial relationships that could be construed as a potential conflict of interest.
