# Supplementary material for: Acclimatization of Rhizophagus irregularis Enhances Zn Tolerance of the Fungus and the Mycorrhizal Plant Partner
Source: Front Microbiol. 2018 Dec 18;9:3156. doi: 10.3389/fmicb.2018.03156 (PMC6305351; doi:10.3389/fmicb.2018.03156)
Supplement: Supplementary file 1 [file Data_Sheet_1.PDF]

Bui and Franken Table S1

Table S1. List of genes used in the study, their putative gene products, accession numbers, primer sequences for qRT-PCR and references for the genes.

Genes	Putative gene products	Acc. No.	Sequences of forward and reverse primer	Reference
RiABC	ATP binding cassette transporter	GQ249346	AGATGACAATCTTAACTGCCCGGAA<br>ACAATGATGGATTTTTTTTCTTTA	Gonzalez-Guerrero et al. 2010a
RiSOD	superoxide dismutase	BI452161	ATTCCACATCCATGAATTCGGTGA<br>GATAGTACGTCCGATTACAGAGT	Gonzalez-Guerrero et al. 2010b
RiMT	methallothionein	BI452270	CATGTGGAAATAAATCTTGTACTTG<br>CAAATGGTTTTATTGAAAAATAAATG	Gonzalez-Guerrero et al. 2007
RiGST	glutathione S-transferase	BM959570	AATGACTATTACAGTTTTCGG<br>GAATTCTTCCGAAAGGATGTTTG	Waschke et al. 2006
RiZnT	Zn transporter	BI452096	CAAGAATTAAAGAGTTATATGGAGT<br>TCGTGATTATCAACTTCATTTGCGA	Gonzalez-Guerrero et al. 2005
RiGRX	glutaredoxin	BM027377	GATGAAGATTCCGAAGGAAGAG<br>CATCACATCCTCCAACGTGTTG	Benabdellah et al. 2009a
RiPDX	pyridoxal 5′-phosphate synthase	AM949787	CCCAAAATTTAAAGGGAGGAGTT<br>GTGCCACTCCACCTTGTATTCT	Benabdellah et al. 2009b
RiTEF1	translation elongation factor EF1-alpha	AJ831587	GCTCCAGGGCATCGTGATTT<br>CCGTCCTTGGAGATACCAGC	Waschke et al. 2006
